# Supplementary figures and images for: ESCRT-0 Is Not Required for Ectopic Notch Activation and Tumor Suppression in Drosophila
Source: PLoS One. 2014 Apr 9;9(4):e93987. doi: 10.1371/journal.pone.0093987 (PMC3981749; doi:10.1371/journal.pone.0093987)

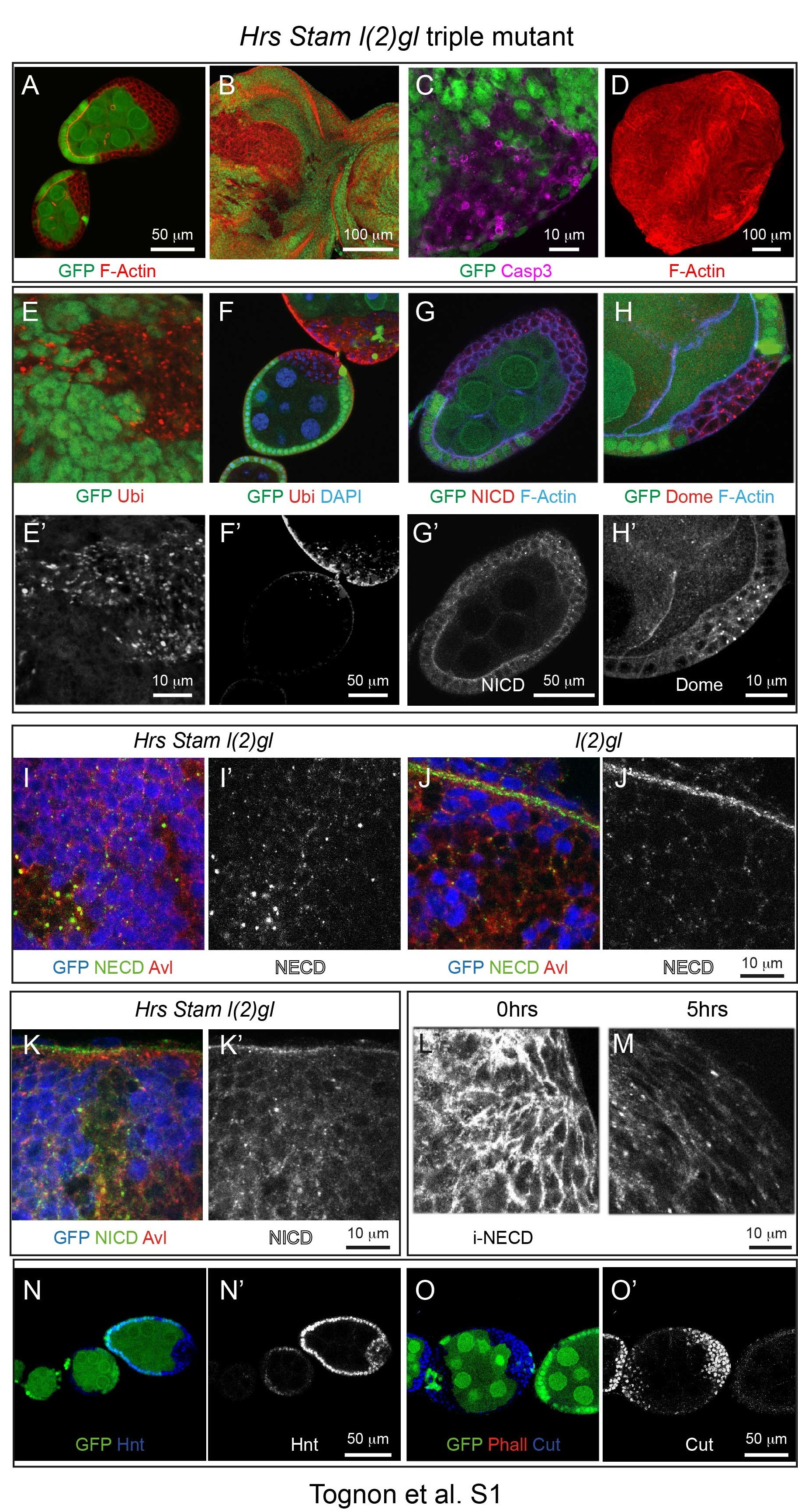

Supplement: Figure S1 — l(2)gl is responsible for the loss of tumor suppression phenotype in triple mutants. (A–B) Epithelial disorganization in Hrs, Stam l(2)gl triple mutant tissues revealed by staining to detect sub-cortical f-Actin. FE cells homozygous for Hrs, Stam l(2)gl (marked by the absence of GFP) in a stage 5–6 egg chamber are misshapen and multilayered. Eye imaginal cells homozygous for the mutations also show mesenchymal-like cells and autonomous disruption of epithelial organization. (C) Mosaic eye imaginal discs stained with antibody anti-activated Caspase 3 to mark apoptotic cells. In Hrs Stam l(2)gl mutant tissue (GFP negative) apoptosis is activated. (D) Eye imaginal disc formed by mutant cells homozygous for Hrs, Stam l(2)gl stained to detect sub-cortical f-Actin show a tumor-like phenotype. (E–H) Hrs Stam l(2)gl mosaic eye and FE cells (marked by the absence of GFP) stained to detect ubiquitin, Notch and the Domeless receptors. Separate channels are shown in E’F’G’H’. Cells homozygous for Hrs Stam l(2)gl show accumulation of ubiquitin, Notch and Domeless intracellularly. (I–K) Co-localization with anti Notch ECD (NECD) or Notch ICD (NICD) and Avl, marking early endosomes, in mosaic eye imaginal discs. Notch ECD is mainly accumulated in early endosomes in GFP-negative Hrs, Stam l(2)gl triple mutant tissue, but not in (2)gl mutant tissue. (L–M) Endocytic trafficking assay with anti-Notch ECD to label Notch at the surface of living Hrs Stam l(2)gl mutant eye disc. Notch receptor fails to be degraded in mutant cells and it remains accumulated intracellularly after 5 hrs after the endo of labeling (0 hrs). (N–O) Mosaic egg chambers at stages 5–7 of oogenesis stained for Hnt and Cut. Hrs Stam l(2)gl homozygous cells are marked by the absence of GFP. In Hrs, Stam l(2)gl triple mutant cells Hnt expression and failure to downregulate Cut expression is visible only in multilayering cells that are likely not reached by the ligand, indicating that Notch activation is not affected i [file pone.0093987.s001.jpg]

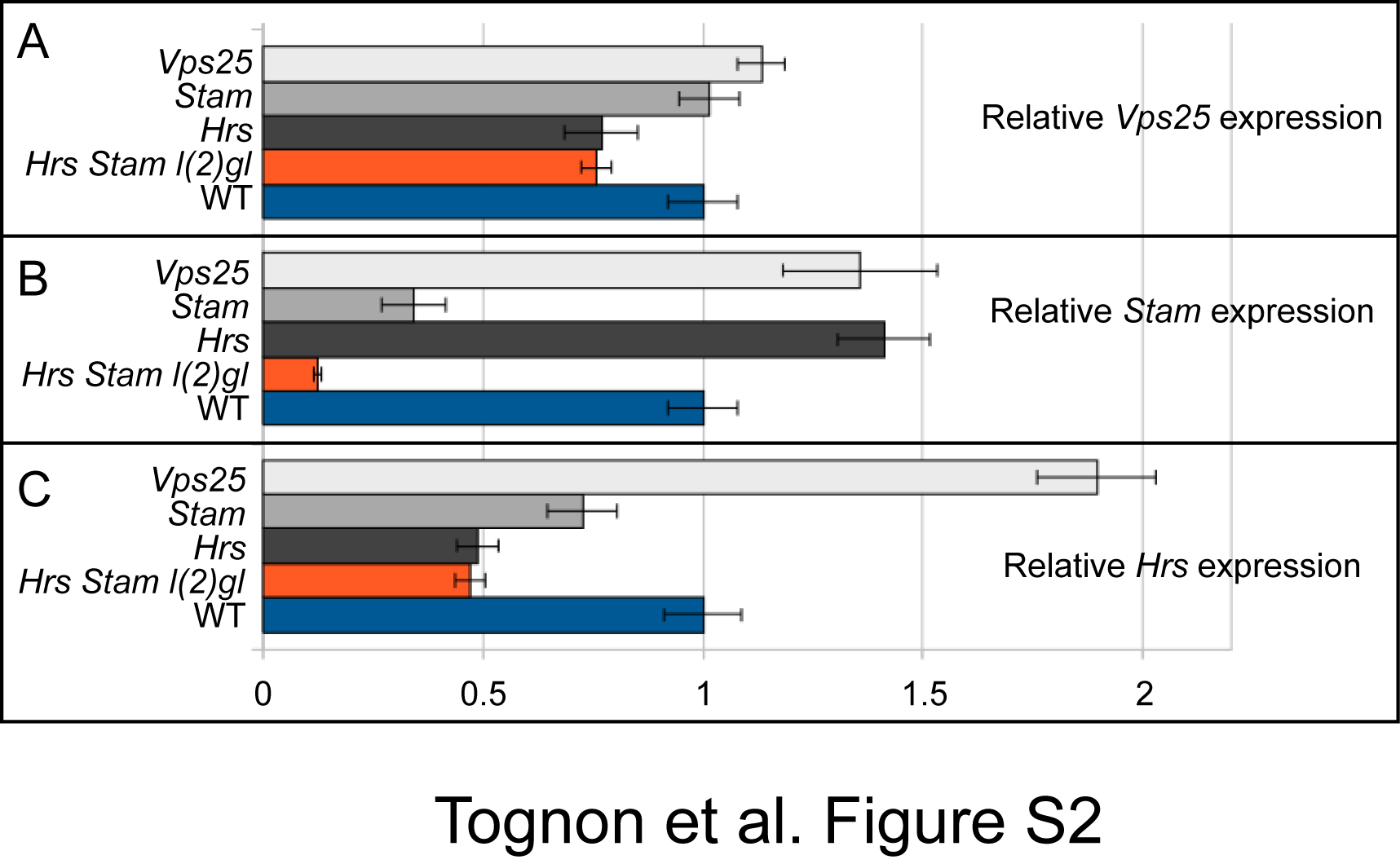

Supplement: Figure S2 — Mutant transcripts for Hrs and Stam are subjected to non sense-mediated decay. Quantitative RT-PCR experiment on mRNA extracted from eye imaginal discs from single Hrs or Stam or double Hrs, Stam or triple Hrs, Stam, l(2)gl mutant tissue compared to control indicates reduction of Hrs or Stam mRNA expression in corresponding mutant extracts. (TIF) [file pone.0093987.s002.tif]
